# Radiomics Analysis of Computed Tomography for Prediction of Thyroid Capsule Invasion in Papillary Thyroid Carcinoma: A Multi-Classifier and Two-Center Study

**DOI:** 10.3389/fendo.2022.849065

**Published:** 2022-05-25

**Authors:** Xinxin Wu, Pengyi Yu, Chuanliang Jia, Ning Mao, Kaili Che, Guan Li, Haicheng Zhang, Yakui Mou, Xicheng Song

**Affiliations:** ^1^ Department of Otorhinolaryngology, Head and Neck Surgery, Yantai Yuhuangding Hospital, Qingdao University, Yantai, China; ^2^ Shandong Provincial Clinical Research Center for Otorhinolaryngologic Diseases, Yantai, China; ^3^ Department of Radiology, Yantai Yuhuangding Hospital, Qingdao University, Yantai, China; ^4^ Big Data and Artificial Intelligence Laboratory, Yantai Yuhuangding Hospital, Qingdao University, Yantai, China

**Keywords:** papillary thyroid carcinoma, radiomics, machine learning, computed tomography, thyroid capsule invasion

## Abstract

**Objective:**

To investigate the application of computed tomography (CT)-based radiomics model for prediction of thyroid capsule invasion (TCI) in papillary thyroid carcinoma (PTC).

**Methods:**

This retrospective study recruited 412 consecutive PTC patients from two independent institutions and randomly assigned to training (n=265), internal test (n=114) and external test (n=33) cohorts. Radiomics features were extracted from non-contrast (NC) and artery phase (AP) CT scans. We also calculated delta radiomics features, which are defined as the absolute differences between the extracted radiomics features. One-way analysis of variance and least absolute shrinkage and selection operator were used to select optimal radiomics features. Then, six supervised machine learning radiomics models (k-nearest neighbor, logistic regression, decision tree, linear support vector machine [L-SVM], Gaussian-SVM, and polynomial-SVM) were constructed. Univariate was used to select clinicoradiological risk factors. Combined models including optimal radiomics features and clinicoradiological risk factors were constructed by these six classifiers. The prediction performance was evaluated using the receiver operating characteristic (ROC) curve, calibration curve, and decision curve analysis (DCA).

**Results:**

In the internal test cohort, the best combined model (L-SVM, AUC=0.820 [95% CI 0.758–0.888]) performed better than the best radiomics model (L-SVM, AUC = 0.733 [95% CI 0.654–0.812]) and the clinical model (AUC = 0.709 [95% CI 0.649–0.783]). Combined-L-SVM model combines 23 radiomics features and 1 clinicoradiological risk factor (CT-reported TCI). In the external test cohort, the AUC was 0.776 (0.625–0.904) in the combined-L-SVM model, showing that the model is stable. DCA demonstrated that the combined model was clinically useful.

**Conclusions:**

Our combined model based on machine learning incorporated with CT radiomics features and the clinicoradiological risk factor shows good predictive ability for TCI in PTC.

## Introduction

Thyroid cancer is the most frequent endocrine malignancy, and papillary thyroid carcinoma (PTC) accounts for about 80%–90% of all cases and the most common histological subtype (1, 2). PTC has a slow disease progression, excellent prognosis, and high survival rate; thus, most patients with PTC are ambivalent in choosing a treatment modality (3). However, surgery is necessary for some rapidly progressing thyroid tumors, such as those with extrathyroidal extension (ETE) and lymph node metastasis (LNM) (4). Therefore, the identification of PTC with ETE or LNM is important.

Thyroid capsule invasion (TCI) is the infiltration of a tumor into the continuous fibrous thyroid capsule without extension into the surrounding soft tissues or the sternothyroid muscle. Indeed, TCI is the premise of ETE (5). Many studies found that TCI is one of the independent risk factors for LNM in the central and lateral cervical regions, whether in papillary thyroid microcarcinoma or PTC (6–9). Mazzaferri suggested that TCI is associated with increased tumor recurrence and distant metastases (10). Early studies demonstrated that TCI contributes to poor prognostic (11–13). Therefore, predicting TCI in PTC is important to assess tumor progression.

However, accurate preoperative assessment of TCI in PTC remains challenging. Although surgical histopathology image analysis serves as the gold standard for diagnosing TCI, it is invasive and cannot predict TCI preoperatively. Computed tomography (CT), a common imaging examination method, has great auxiliary value in preoperatively evaluating and determining the extent, localization, and lymph node (LN) status of the tumor (14, 15).

However, up to now, most diagnostic information from CT is based on visual inspection by a radiologist, who may miss critical diagnostic information. Thus, conventional CT is still not effective in diagnosing TCI. Radiomics, which is the quantitative analysis of a large amount of data in medical images using computer technology, has received increasing attention because of its improved diagnosis and prediction accuracy (16, 17). Moreover, no studies have been conducted to predict TCI in PTC using radiomics analysis.

Therefore, this study aimed to propose and validate a machine learning-based method to preoperatively predict TCI in PTC by combining CT-based radiomics features and clinicoradiological characteristics.

## Materials and Methods

### Patients

This retrospective study was approved by the clinical institutional review boards of the two selected institutions, and patient informed consent was waived. A total of 412 consecutive patients were recruited. The 379 eligible patients recruited from Yantai Yuhuangding Hospital (Institution I) from March 2018 to March 2020 were divided into the training cohort (n=265) and internal test cohort (n=114) at a ratio of 7:3. The 33 eligible patients recruited from Qilu Hospital of Shandong University (Institution II) from September 2020 to December 2020 served as the external test cohort.

**Figure 1:**
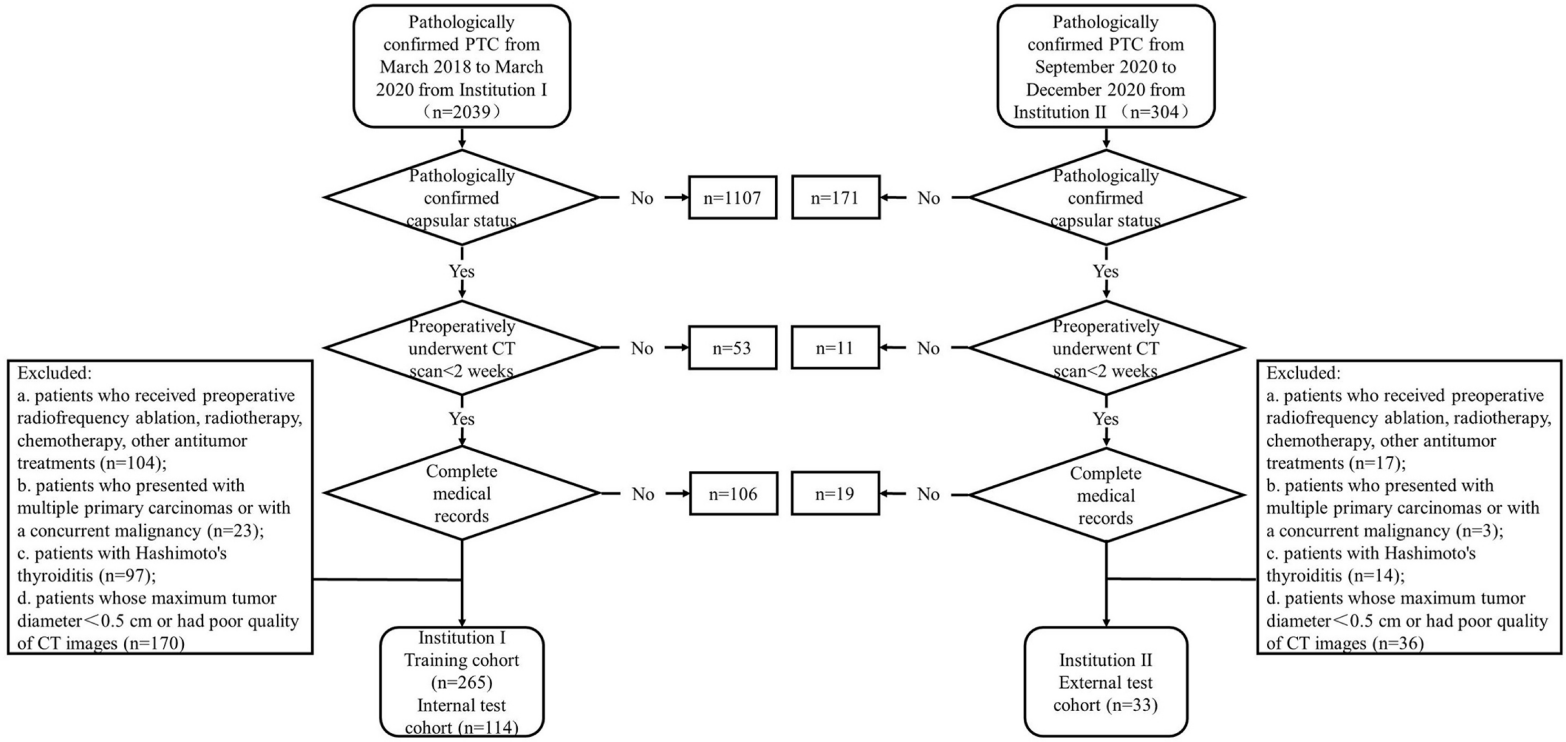
The patient recruitment pathway in the two-center study.

The inclusion criteria were as follows: (a) patients who preoperatively underwent non-enhanced and contrast-enhanced CT scans for <2 weeks; (b) patients who had pathologically confirmed PTC after surgical resection; (c) patients who had pathologically confirmed capsular status after surgery; and (d) patients with well-preserved clinical data, imaging data, and pathological specimens. The exclusion criteria were as follows: (a) patients who received preoperative radiofrequency ablation, radiotherapy, chemotherapy, or other antitumor treatments; (b) patients who received prior treatment in other institutions; (c) patients who presented with multiple primary carcinomas or concurrent malignancy; (d) patients with Hashimoto’s thyroiditis; (e) patients whose maximum tumor diameter < 0.5 cm or had poor quality of CT images. The patient recruitment pathway is depicted in **Figure 1**.

### Clinicoradiological Characteristics

Clinicoradiological characteristics, including age, sex, thyroid-stimulating hormone (TSH), tumor location (left lobe, right lobe, and isthmus), CT-reported maximum tumor diameter (CT-MTD), CT-reported TCI (positive and negative), and CT-reported LN status (positive, negative, and suspicious) were collected. A laboratory analysis of TSH was performed <2 weeks before surgery. The preoperative CT scans of all patients were retrospectively reviewed and verified by two radiologists (Radiologist 1 has 12 years of experience in thyroid imaging and Radiologist 2 has 10 years of experience in thyroid imaging) who did not have knowledge of the histopathological findings.

The radiologists recorded the tumor location, CT-MTD, TCI status, and LN status in the CT images. CT-MTD was recorded as the mean value. In CT images, tumor with an irregular shape, tumor that breaks through the thyroid capsule, a contact area with the thyroid margin > 25% of the tumor circumference, and the presence of a reduction/blurring of the focal extent after enhancement were considered to have CT-reported TCI (positive). Based on the National Comprehensive Cancer Network guidelines (18), relevant literature (14, 19), and diagnostic experience, the CT diagnostic criteria for LNM in patients with PTC were as follows: (a) LN maximal short-axis diameter > 10 mm; (b) round or irregular shape; (c) rough margin, fuzzy boundary, and/or invasion into adjacent tissues; (d) calcification or cystic and/or necrotic change; (e) strong enhancement (similar to or stronger than that of the pharyngeal mucosa); and (f) heterogeneous enhancement. A patient’s CT-reported LN status was classified as positive if one or more LNs found in the CT images met any one of the above criteria. LN was considered suspicious when LN did not meet the above criteria but had a short-axis diameter > 5 mm at cervical region VI (20, 21). A LN that did not meet the above criteria was considered to have a negative LN statis. Any disagreements were resolved by consensus or the consultation with a third radiologist who had 20 years of experience. κ-statistic was calculated to determine the inter-observer agreement between two radiologists, where 0<κ≤0.4 indicates poor agreement, 0.4<κ<0.75 indicates good agreement, and 0.75≤κ<1 indicates high agreement.

### CT Acquisition Parameters

Preoperative non-contrast (NC) and contrast-enhanced CT scans were performed for each patient at the two institutions. Institution I performed CT scans using two CT scanners: a 64-slice spiral CT scanner (Siemens, Germany) or a 256-slice spiral CT scanner (Philips, Netherlands). Institution II performed CT scans using four CT scanners: a 16-slice spiral CT scanner (Siemens, Germany), a 64-slice spiral CT scanner (GE, USA), a 64-slice spiral CT scanner (Toshiba, Japan), or a 256-slice spiral CT scanner (Philips, Netherlands).

The parameters for the CT scan were as follows: tube voltage, 100 or 120 kV; tube current, 180–400 mA·s; reconstruction section thickness, 1.25–5.00 mm; pitch, 0.97–1.5; and matrix, 512×512. The scan range was from the skull base to the subclavian region. After routine plain CT scans, contrast-enhanced CT scans were performed after a delay of 20–30 s (arterial phase [AP]) following an intravenous administration of 80–100 mL of iodinated nonionic contrast agent at a rate of 3.0–3.5 mL/s using a high-pressure syringe. The nonionic contrast agent used was iohexol (Yangtze River, China; GE Healthcare, Ireland).

### Image Segmentation

**Figure 2:**
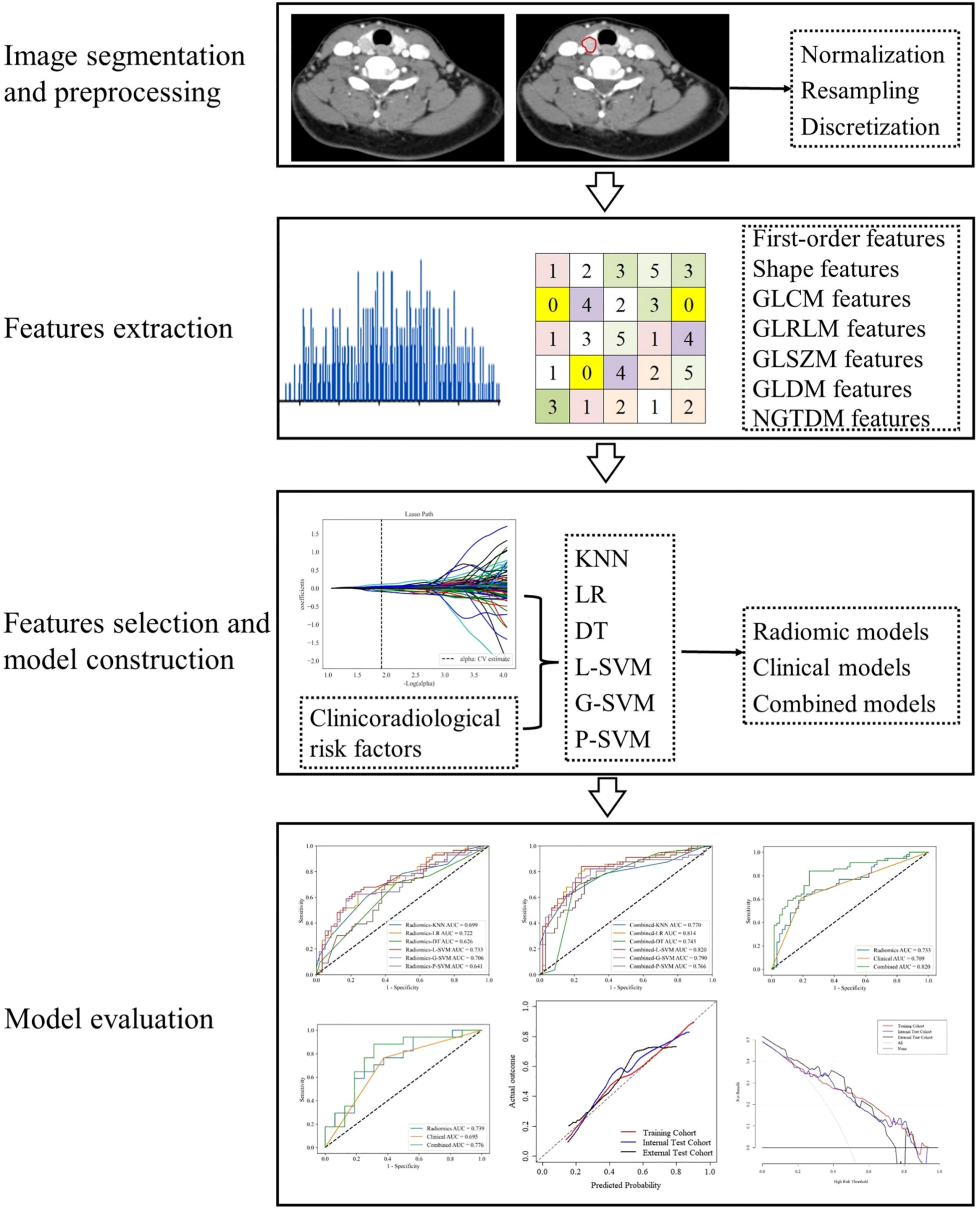
Workflow of data analysis. The workflow illustrates image segmentation and preprocessing, radiomics features extraction and selection, models construction and evaluation.

All CT images were retrieved from the Picture Archiving and Communication System with the data format of Digital Imaging and Communications in Medicine and then loaded into a radiomics cloud platform (http://radcloud.cn/) for manual segmentation. All clinical and pathological information was hidden when the data was uploaded to the platform. Volume of interest (VOI) segmentation was manually drawn slice by slice on the entire tumor’s boundary by radiologist 1. A sample of the segmentation process is presented in **Figure 2**.

### Image Preprocessing

The image data analyzed in this study were obtained from various CT scanners. As highlighted by many previous studies, voxels were diverse if acquired from different scanners (21–23). The diversity of voxels leads to variability in feature values. Therefore, the images were preprocessed to extract robust radiomics features as follows: (1) voxel density normalization ( ± 3σ technique), (2) voxel size resampling (1×1×1 mm^3^, and (3) voxel intensity discretization (64 bins).

### Radiomics Feature Extraction and Selection

Radiomics features were automatically extracted from the VOIs of the non-contrast (NC) and arterial phase (AP) images of each patient based on the “pyradiomics” package in Python (version 3.6). Delta radiomics features (AP−NC), which are defined as the absolute differences between the radiomics features extracted from AP and NC phases, were also computed. Before the radiomics features selection process, Z-scores were used to standardize each radiomics feature to eliminate the differences between features. The features in the internal test and external test cohorts were normalized according to the mean and standard deviation (SD) of the training cohort.

The extracted features were divided into four categories: (1) first-order statistics features, which describe the distribution of voxel intensities within the image region defined through commonly used and basic metrics, such as mean, maximum, minimum, median, energy, entropy, skewness, and kurtosis; (2) shape features, which reflect the shape and size of the region, such as surface area, sphericity, compactness, and maximum diameter; (3) texture features, which were calculated from the Gray-level Co-occurrence Matrix (GLCM), Gray-level Run Length Matrix (GLRLM), Gray-level Size Zone Matrix (GLSZM), Gray-level Dependence Matrix (GLDM), and neighborhood gray tone difference matrix (NGTDM); and (4) higher-order statistical features, which include the first-order statistics and texture features obtained through the wavelet transformation and filter transformation of the original images, such as logarithm, square, square root, wavelet, exponential, and gradient.

Sixty patients were randomly selected from the training cohort by statistical software to evaluate the inter-and intra-observer agreement of the extracted radiomics features. Radiologist 2 used the same tool and method for tumor segmentation. After 3 months, tumor segmentation was repeated by radiologist 1. Inter- and intra-correlation coefficients (ICCs) were calculated to determine the reproducibility of radiomics features. ICCs > 0.75 represent good agreement (24, 25).

The following features selection strategies were used to reduce the dimensionality and select the best subset of features. First, features with ICCs > 0.75 were retained. Second, features with p < 0.05 were selected after one-way analysis of variance (ANOVA). Then, the least absolute shrinkage and selection operator algorithm (LASSO) with penalty tuning conducted by 10-fold cross-validation was applied to select the key radiomics features with nonzero coefficients.

### Clinicoradiological Risk Factor Selection

Univariate analysis was applied to the clinicoradiological characteristics of the training cohort to select the clinicoradiological risk factor associated with TCI. Odds ratios (ORs) as estimates of relative risk with 95% confidence intervals (CIs) were calculated for each risk factor.

### Model Construction

Most previous thyroid-related radiomics studies used logistic regression (LR) as a classifier (26–28). K-nearest neighbor (KNN), decision tree (DT), linear support vector machine (L-SVM), Gaussian support vector machine (G-SVM), and polynomial support vector machine (P-SVM) are also commonly used machine learning classifiers in radiomics studies. In this study, based on these six classifiers, models based on the optimal radiomics features (radiomics model), the clinicoradiological risk factor (clinical model), and combined model were constructed, respectively. LR, KNN, DT, and SVM were performed using Python (version 3.6) with scikit-learn package (https://scikit-learn.org/).

In the training process, the hyperparameters of each classifier were tuned by an iterative grid search procedure to avoid overfitting and maximize the performance of the model. A 5-fold cross-validation was applied to tune the model parameters.

### Model Evaluation

All models were trained in the training cohort, performance was assessed by 5-fold cross-validation, and the process was repeated 10 times to calculate the mean of performance estimates. The prediction performance was evaluated by using receiver operating characteristic (ROC) curve and calculating the area under the ROC curve (AUC). The calibration curves of the optimal combined model were used to evaluate the agreement between the observed results and the predicted probabilities. Decision curve analysis (DCA) was used to calculate the net benefits for threshold probabilities determine the clinical usefulness of the optimal combined model.

### Statistical Analysis

Normally distributed data are expressed as mean ± SD, and non-normally distributed data are presented as median (interquartile range). Continuous characteristics were compared by two-sample t-test or Mann-Whitney U test, whereas categorical characteristics were analyzed by chi-square test or Fisher’s exact test. Statistical analysis was performed in R software (version 4.0.3) and Python (version 3.6). “rms,” “rmda,” and “irr,” packages in R were used. Python scikit-learn package was employed to select radiomics features and construct and evaluate models. “selectKbest,” “LassoCV,” “LogisticRegression,” “svm,” “neighbors,” “tree,” and “roccurve,” packages were used. All statistical tests were two-sided, and p<0.05 was considered a statistically significant difference.

## Results

### Patients and Clinicoradiological Characteristics

A total of 412 patients were divided into pTCI+ (pathological positive TCI) and pTCI− (pathological negative TCI) based on postoperative pathological findings. Inter-observer agreement for CT-reported TCI was good (κ=0.734, 95% confidence interval [CI]=0.658–0.829). Inter-observer agreement for CT-reported LN status was high (κ=0.819, 95% CI=0.776–0.900). The clinicoradiological characteristics of patients in the training, internal test, and external test cohorts are summarized in **Table 1**.

**Table 1:** Clinicoradiological characteristics of the training, internal test, and external test cohorts.

	Training cohort (n=265)			Internal test cohort (n=114)			External test cohort (n=33)		
	pTCI + (n=130)	pTC I− (n=135)	*p* value	pTCI + (n=56)	pTCI − (n=58)	*p* value	pTCI + (n=17)	pTCI − (n=16)	*p* value
**Gender**			0.692			0.035			0.389
Male	30/23.1	35/25.9		38/67.9	50/86.2		4/23.5	7/43.8	
Female	100/76.9	100/74.1		18/32.1	8/13.8		13/76.5	9/56.3	
Age (years)^a^	45.52 ± 11.88	43.87 ± 11.14	0.246	46.20 ± 11.01	44.60 ± 11.77	0.635	46.05 ± 13.41	44.88 ± 11.77	0.789
TSH (mIU/L)^a^	2.60 ± 2.76	2.44 ± 1.18	0.551	2.41 ± 1.70	2.38 ± 1.17	0.915	2.28 ± 1.35	2.04 ± 0.92	0.927
CT-MTD (cm)^a^	1.01 ± 0.58	0.99 ± 0.55	0.807	1.04 ± 0.48	0.95 ± 0.44	0.345	1.32 ± 0.50	1.08 ± 0.65	0.242
**Location**			<0.001			0.451			<0.001
Left	66/50.8	60/44.4		27/48.2	23/39.7		5/29.4	3/18.75	
Right	59/45.4	73/54.1		29/51.8	35/60.3		12/70.6	12/75.0	
Isthmus	5/3.8	2/1.5		0	0		0/0.0	1/6.25	
**CT-reported TCI**			<0.001			<0.001			0.037
Yes	80/61.5	20/14.8		34/60.7	11/19.0		13/76.5	6/37.5	
No	50/38.5	115/85.2		22/39.3	47/81.0		4/23.5	10/62.5	
**CT-reported LN status**			<0.001			<0.001			0.001
Positive	29/22.3	17/12.6		11/19.6	10/17.2		7/41.2	4/25.0	
Negative	79/60.8	92/68.1		32/57.2	39/67.3		9/52.9	12/75.0	
Suspicious	22/16.9	26/19.3		13/23.2	9/15.5		1/5.9	0/0.0	

The data are displayed as n/% except otherwise noted.

a Mean ± standard deviation

pTCI+, pathologically positive thyroid capsule invasion; pTCI−, pathologically negative thyroid capsule invasion; TSH, thyroid-stimulating hormone; CT, computed tomography; CT-MTD, CT-reported maximum tumor diameter;TCI, thyroid capsule invasion; LN, lymph node.

### Radiomics Features Extraction and Selection

**Figure 3:**
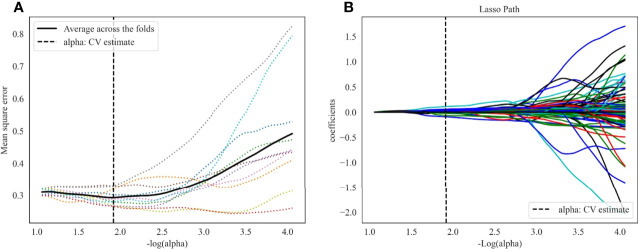
LASSO algorithm for radiomics features selection. **(A)** Mean square error path using 10-fold cross validation. **(B)** LASSO coefficient profiles of the radiomics features.

1409 radiomics features were extracted from each CT phase, followed by a calculation of delta radiomics features. A total of 4227 (1409 × 3) radiomics features were extracted from each patient. The inter-observer ICCs calculated based on radiologist 1’s first-extracted features and those of radiologist 2 ranged from 0.766 to 0.897. The intra-observer ICCs calculated based on radiologist 1’s twice features extraction ranged from 0.821 to 0.943. These results showed that features extraction within and between observers had good repeatability. Then, 640 features (p<0.05) were further selected by ANOVA. Finally, 23 optimal radiomics features were selected through the LASSO method with all features from the NC (6 first-order statistical feature, 4 shape-based feature, and 13 textural features [GLDM, n = 4; GLRLM, n = 3; GLSZM, n = 6]; **Figure 3**). The most predictive radiomics features are described in detail in **Supplementary Material Table S1**.

### Clinicoradiological Risk Factors Selection

In the training cohort, CT-reported TCI (OR=1.80, 95% CI 1.60–2.02, p < 0.001) was identified as the clinicoradiological risk factor of TCI in PTC (**Table 2**).

**Table 2:** Univariate analysis of clinicoradiological characteristics in the training cohort.

	Univariate analysis	
	OR (95%CI)	*p* value
**Sex**	1.04 (0.90-1.20)	0.592
**Age (years)**	1.00 (0.99-1.01)	0.245
**TSH (mIU/L)**	1.01 (0.98-1.04)	0.527
**CT-MTD (cm)**	1.01 (0.91-1.13)	0.807
**Location**	0.97 (0.87-1.08)	0.558
**CT-reported TCI**	1.80 (1.60-2.02)	<0.001
**CT-reported LN status**	1.02 (0.94-1.10)	0.601

TSH, thyroid-stimulating hormone; CT, computed tomography; CT-MTD, CT-maximum tumor diameter; TCI, thyroid capsule invasion; LN, lymph node; OR, odds ratio; CI, confidence interval.

### Predictive Performance of Models

**Figure 4:**
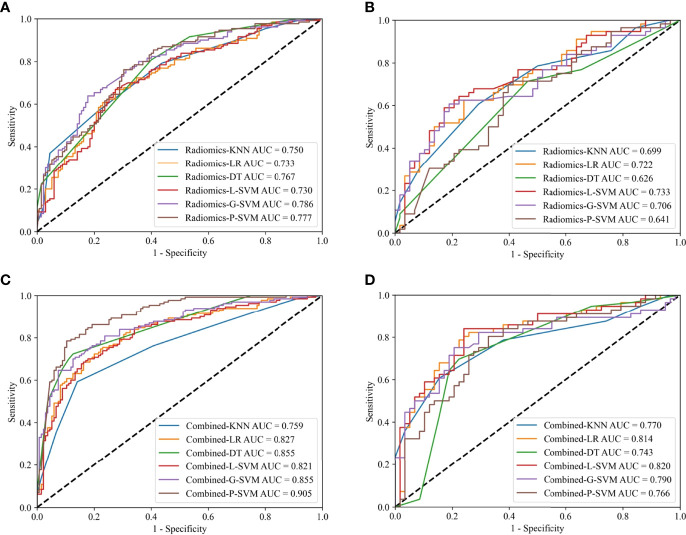
ROC curves for the radiomics models in the training **(A)** and internal test **(B)** cohorts; ROC curves for the combined models in the training **(C)** and internal test **(D)** cohorts.

Radiomics models based on the optimal radiomics features alone were constructed. In the training cohort, radiomics-G-SVM model achieved the most satisfactory results with AUC 0.786 (95%CI 0.736–0.832). In the internal test cohort, radiomics-L-SVM model achieved the most satisfactory results with AUC 0.733 (95%CI 0.654–0.812) (**Figures 4A, B**).

Clinical model based on the clinicoradiological risk factor (CT-reported TCI) alone was constructed. The AUC of the clinical model was 0.734 (95% CI 0.688-0.776) and 0.709 (95% CI 0.649-0.783) in the training and internal test cohorts, respectively.

Combined models that comprise the optimal radiomics features and the clinicoradiological risk factor were constructed. In the training cohort, combined-P-SVM model achieved the most satisfactory results with AUC 0.905 (95%CI 0.871–0.934). In the internal test cohort, the highest AUC was 0.820 (95%CI 0.758–0.888) in combined-L-SVM model (**Figures 4C, D**). The parameters for the models’ predictive performances were summarized in detail in **Supplementary Material Table S2**.

**Figure 5:**
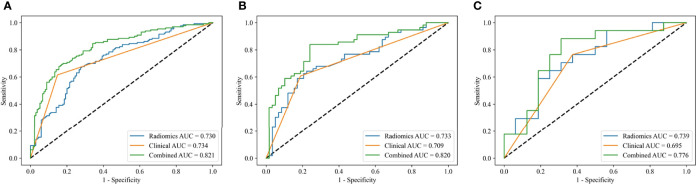
ROC curves of the radiomics models, clinical models and combined models constructed by L-SVM in the training **(A)**, internal test **(B)** and external test **(C)** cohorts.

The three models constructed by the L-SVM classifier to be evaluated in the training, the internal test, and the external test cohorts, respectively (**Figure 5**). The combined-L-SVM model performed better than radiomics and clinical models. Among them, in the external test cohort, the AUC was 0.776 (0.625–0.904) showing that the model is stable.

**Figure 6:**
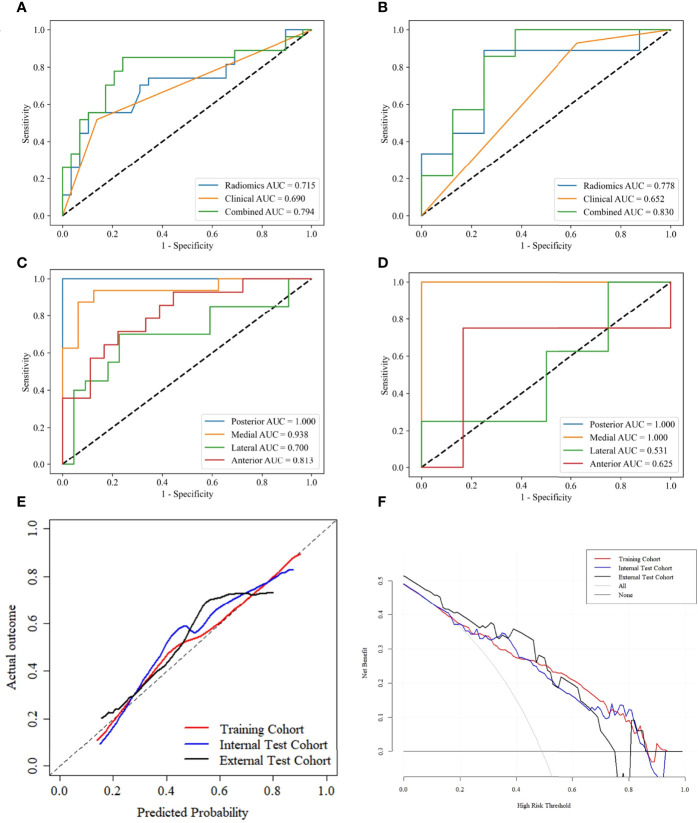
ROC curves of PTCs close to thyroid capsule in the internal test **(A)** and external test **(B)** cohorts. ROC curves for predicting lesions close to different adjacent structures using the combined L-SVM model in the internal test **(C)** and external test **(D)** cohorts. Calibration curves of the combined-L-SVM models in the training, internal test and external test cohorts **(E)**. DCA of the combined-L-SVM models in the training, internal test and external test cohorts **(F)**.

The lesions close to the thyroid capsule was examined using the combined-L-SVM model. The AUC was 0.794 (95% CI 0.701-0.912) and 0.830 (95% CI 0.620-0.983) in internal test and external test cohorts, respectively (**Figures 6A, B**).

We grouped lesions locations in the internal test and external test cohorts according to different adjacent structures: posterior (esophagus), medial (trachea), lateral (carotid sheath), anterior (strap muscle), and performed stratified analysis using the combined-L-SVM model. In both cohorts, the model performed relatively well in lesions close to the medial and posterior, with AUCs of 0.938 and 1.000 in the internal test cohort and 1.000 and 1.000 in the external test cohort, respectively (**Figures 6C, D**).

The calibration curves of the combined-L-SVM model indicated good calibration between predictive outcome and observation in the training, internal test, and external test cohorts (**Figure 6E**). The DCA showed that the combined-L-SVM model to predict TCI could provide more benefit than the treat-all-patients scheme or the treat-all-none scheme, when the threshold probability range from 0.20–0.86 in the training and internal test cohorts, 0.20–0.74 in the external test cohort (**Figure 6F**).

## Discussion

We established combined models based on machine learning incorporated with CT radiomics features and the clinicoradiological risk factor to individualize the prediction of TCI in PTC. Moreover, we tested the models using internal and independent external test cohorts. The combined-L-SVM model demonstrated good predictive ability and clinical usefulness in the training and test cohorts, which indicates that the combined model could be an effective, non-invasive, and safe tool for preoperative prediction of TCI in PTC.

Akbulut et al. (29) found that patients with TCI are younger on average than those with non-invasive tumors (p=0.035). Luo et al. (30) suggested that TCI and patient age do not correlate (p=0.863). Our study fits with the findings of Luo et al., that is, age may not be associated with TCI (p=0.245). A consensus on whether TSH level is an independent predictive factor of TCI in PTC has not yet been established. A previous study reported that patients with TCI have remarkably higher TSH levels than those without TCI (31). However, some studies believed that TSH level is not a predictor of tumor aggressiveness. In our study, TSH level may not be associated with TCI (p=0.527). Previous studies have shown that PTC with TCI is associated with location and tumor size. Pontieri G et al. (32) and Zhang et al. (33) reported that PTC localized in the isthmus had a high rate of TCI. Furlan et al. (34) reported that PTC with capsular invasion is associated with larger tumors than PTC without capsular invasion. However, our study did not obtain the above results in terms of the correlation between capsule invasion and tumor size/location (P=0.807/0.558). The reasons may be the differences in the selection of sample and the size of sample. Besides, Luo et al. (30) revealed that there was no correlation between LNM and with/without TCI of PTC, which was similar to our study (P=0.601).

Notably, many previous studies on radiomics have only provided an internal test cohort, and all data were obtained from a single piece of equipment in a single center. However, studies have confirmed that equipment from different manufacturers leads to differences in scanning parameter settings and post-processing reconstruction algorithms, which result in remarkable differences in radiomics features (23, 35, 36). Although some studies have achieved good results, the generalizability of the models was not confirmed because the studies were conducted in single centers. Therefore, single-center studies have their limitations (37). This problem was addressed in the present study by including an external test cohort to assess model performance. In addition, image preprocessing was performed before feature extraction to reduce the dependency on image specifications. Our results showed that the prediction performance of the model in the external test cohort was still good, which illustrates the generalizability of our model.

The model construction methods used in many studies were relatively simple, and the differences in models constructed by different classifiers were not adequately discussed. For example, in our previous study, we only used the LR-based model to identify <1 cm benign and malignant thyroid lesions, and the model performed excellently in the training and test sets with AUCs of 0.853 and 0.851, respectively (38). However, Lambin et al. (16) showed that studies on radiomics should use multiple machine learning methods. LR is a regression method that eliminates the selected features with little contribution to the linear model. However, the potential relationship between the radiomics features and lesions is complex and may be non-linear during radiomics analysis. Masataka et al. (39) applied six machine learning classifiers to distinguish uterine sarcomas from leiomyomas using image texture analysis, and the resulting AUCs ranged from 0.68 to 0.93. These results suggest that the diagnostic performance of radiomics analysis is highly dependent on the selection of machine learning classifiers. Six types of supervised machine learning classifiers (i.e., LR, KNN, DT, L-SVM, G-SVM, and P-SVM) were used in model construction to improve the performance of the models in the current study. The results showed that the L-SVM-based model had the best performance. SVM is a powerful and robust machine learning classifier that has been used to solve a range of high-dimensional, non-linear problems (40).

Our study was performed on the VOIs of NC and AP images rather than on the VOI of a single CT scan. At the same time, the delta radiomics features of tumors were also calculated. Interestingly, only features from NC images were used in our model, which suggests that these features may be more helpful in identifying thyroid TCI than AP and delta radiomics features. In our previous study, most of the radiomics features used in identifying <1 cm benign and malignant thyroid lesions were also extracted from NC images (38).

Our study has several limitations. First, although this study was based on two centers (both from Northern China), prospective studies with more centers should be involved to provide more diverse data to interpret tumor heterogeneity and construct models with greater stability and accuracy. Second, fully automatic or semi-automatic image segmentation techniques are still immature for the irregular shape and uncertain contour of thyroid tumors; therefore, automatic and semi-automatic segmentation techniques will be further explored in our future study. Third, previous studies have suggested that TCI may be closely associated with B-Raf proto-oncogene serine/threonine kinase (*BRAF*) mutations (5, 41). However, this variable was not included in our study because of a lack of *BRAF* information in some patients. In addition, although stratified analysis of tumor location by posterior, medial, lateral, and anterior revealed that the model performed relatively well at posterior and medial locations in this study, the results may not be very stable due to the small sample size of the subgroup. Finally, although radiomics features can manifest tumor heterogeneity, tumor heterogeneity may be comprehensively quantified through a combination of pathological imaging, proteomics, and genomic sequencing.

In conclusion, our combined model based on machine learning incorporated with CT radiomics features and the clinicoradiological risk factor shows good predictive ability for TCI in PTC. Further studies using large sample size, multiple centers, multi-modes, different ethnic groups, and different geographical locations should be performed to improve the model efficiency.

## Data Availability Statement

The raw data supporting the conclusions of this article will be made available by the authors, without undue reservation.

## Ethics Statement

The studies involving human participants were reviewed and approved by Yantai Yuhuangding Hospital and Qilu Hospital of Shandong University. Written informed consent for participation was not required for this study in accordance with the national legislation and the institutional requirements.

## Author Contributions

XW, PY and CJ contributed to the data analysis and the manuscript preparation. XS, YM, NM, XW contributed to the conception and design of the study. PY, KC, GL, HZ contributed to data acquisition and analysis. PY, XS and YM contributed to the manuscript revision. All authors contributed to the article and approved the submitted version.

## Funding

This work was supported by Taishan Scholars Project (No. ts20190991).

## Conflict of Interest

The authors declare that the research was conducted in the absence of any commercial or financial relationships that could be construed as a potential conflict of interest.

## Publisher’s Note

All claims expressed in this article are solely those of the authors and do not necessarily represent those of their affiliated organizations, or those of the publisher, the editors and the reviewers. Any product that may be evaluated in this article, or claim that may be made by its manufacturer, is not guaranteed or endorsed by the publisher.

## Abbreviations

AP, artery phase; AUC, area under the receiver operating characteristic curve; CI, confidence interval; CT, computed tomography; CT-MTD, CT-reported maximum tumor diameter; DCA, decision curve analysis; DT, decision tree; ETE, extrathyroidal extension; G-SVM, Gaussian support vector machine; ICCs, inter- and intra-correlation coefficients; KNN, k-nearest neighbor; LASSO, least absolute shrinkage and selection operator; LN, lymph node; LNM, lymph node metastasis; LR, logistic regression; L-SVM, linear support vector machine; NC, non-contrast CT; PTC, papillary thyroid carcinoma; P-SVM, polynomial support vector machine; ROC, receiver operating characteristic; VOI, volume of interest; TCI, thyroid capsule invasion; TSH, thyroid-stimulating hormone.
