# Supplementary material for: Radiomics Analysis of Computed Tomography for Prediction of Thyroid Capsule Invasion in Papillary Thyroid Carcinoma: A Multi-Classifier and Two-Center Study
Source: Front Endocrinol (Lausanne). 2022 May 25;13:849065. doi: 10.3389/fendo.2022.849065 (PMC9174423; doi:10.3389/fendo.2022.849065)
Supplement: Supplementary file 1 [file Table_1.docx]

Radiomics Analysis of Computed Tomography for Prediction of Thyroid Capsule Invasion in Papillary Thyroid Carcinoma: A Multi-classifier and Two-center Study

S1. The most predictive radiomics features.

| **Radiomics Features** | **Feature weights** |
| --- | --- |
| Sphericity_original_shape_NC | 0.005294021 |
| Maximum3DDiameter_original_shape_NC | -0.014692565 |
| Maximum2DDiameterColumn_original_shape_NC | -0.094220179 |
| 10Percentile_original_firstorder_NC | -0.007536027 |
| Flatness_original_shape_NC | 0.055555634 |
| DependenceNonUniformityNormalized_original_gldm_NC | 0.110569356 |
| RunLengthNonUniformityNormalized_original_glrlm_NC | 2.12E-05 |
| ZoneVariance_original_glszm_NC | 0.01206185 |
| ZonePercentage_original_glszm_NC | -0.063914501 |
| Skewness_logarithm_firstorder_NC | -0.004961792 |
| DependenceNonUniformityNormalized_logarithm_gldm_NC | 0.007485853 |
| ZonePercentage_logarithm_glszm_NC | -3.35E-07 |
| RobustMeanAbsoluteDeviation_exponential_firstorder_NC | 0.035152841 |
| RunLengthNonUniformityNormalized_logarithm_glrlm_NC | 0.044191088 |
| ZoneVariance_logarithm_glszm_NC | 3.95E-06 |
| DependenceNonUniformityNormalized_exponential_gldm_NC | 0.014197326 |
| ZonePercentage_exponential_glszm_NC | -0.030593733 |
| RunLengthNonUniformityNormalized_exponential_glrlm_NC | 0.026726735 |
| ZoneVariance_exponential_glszm_NC | 2.16E-07 |
| Skewness_gradient_firstorder_NC | 0.035658654 |
| Minimum_gradient_firstorder_NC | 0.057231816 |
| DependenceNonUniformityNormalized_gradient_gldm_NC | 0.003764186 |
| Energy_gradient_firstorder_NC | 0.01192214 |

Abbreviations: NC, non-contrast CT; gldm, gray level dependence matrix; glrlm, gray level run length matrix; glszm, gray level size zone matrix.

S2. Performance comparison of different models in the training and internal test cohorts.

| Model | Training cohort | | | |  | Internal test cohort | | | |
| --- | --- | --- | --- | --- | --- | --- | --- | --- | --- |
|  | AUC | Accuracy | Sensitivity | Specificity |  | AUC | Accuracy | Sensitivity | Specificity |
| Radiomics-KNN | 0.750 | 0.679 | 0.569 | 0.785 |  | 0.699 | 0.658 | 0.607 | 0.707 |
|  | (0.695-0.793) | (0.619-0.735) | (0.480-0.655) | (0.705-0.849) |  | (0.613-0.776) | (0.563-0.744) | (0.468-0.732) | (0.571-0.815) |
| Radiomics-LR | 0.733 | 0.694 | 0.662 | 0.726 |  | 0.722 | 0.649 | 0.661 | 0.638 |
|  | (0.680-0.783) | (0.635-0.749) | (0.573-0.741) | (0.641-0.797) |  | (0.641-0.800) | (0.554-0.736) | (0.521-0.778) | (0.501-0.757) |
| Radiomics-DT | 0.767 | 0.611 | 0.223 | 0.985 |  | 0.626 | 0.544 | 0.089 | 0.983 |
|  | (0.720-0.806) | (0.550-0.670) | (0.157-0.306) | (0.942-0.997) |  | (0.546-0.698) | (0.448-0.637) | (0.033-0.204) | (0.895-0.999) |
| Radiomics-L-SVM | 0.730 | 0.694 | 0.669 | 0.719 |  | 0.733 | 0.675 | 0.679 | 0.672 |
|  | (0.679-0.780) | (0.635-0.749) | (0.581-0.748) | (0.634-0.791) |  | (0.654-0.812) | (0.581-0.760) | (0.539-0.794) | (0.535-0.786) |
| Radiomics-G-SVM | 0.786 | 0.728 | 0.631 | 0.822 |  | 0.706 | 0.675 | 0.518 | 0.828 |
|  | (0.736-0.832) | (0.671-0.781) | (0.541-0.712) | (0.750-0.881) |  | (0.617-0.786) | (0.581-0.760) | (0.382-0.652) | (0.701-0.910) |
| Radiomics-P-SVM | 0.777 | 0.725 | 0.754 | 0.696 |  | 0.641 | 0.588 | 0.500 | 0.672 |
|  | (0.727-0.824) | (0.667-0.777) | (0.669-0.823) | (0.610-0.771) |  | (0.545-0.722) | (0.492-0.679) | (0.365-0.635) | (0.535-0.786) |
| Clinical | 0.734 | 0.736 | 0.615 | 0.852 |  | 0.709 | 0.711 | 0.607 | 0.810 |
|  | (0.688-0.776) | (0.678-0.788) | (0.526-0.698) | (0.778-0.905) |  | (0.649-0.783) | (0.618-0.792) | (0.468-0.732) | (0.682-0.897) |
| Combined-KNN | 0.759 | 0.649 | 0.354 | 0.933 |  | 0.770 | 0.667 | 0.357 | 0.966 |
|  | (0.708-0.801) | (0.588-0.706) | (0.273-0.443) | (0.874-0.967) |  | (0.691-0.838) | (0.572-0.752) | (0.237-0.497) | (0.870-0.994) |
| Combined-LR | 0.827 | 0.759 | 0.746 | 0.770 |  | 0.814 | 0.781 | 0.821 | 0.741 |
|  | (0.781-0.868) | (0.702-0.809) | (0.661-0.817) | (0.689-0.836) |  | (0.749-0.882) | (0.694-0.853) | (0.692-0.907) | (0.607-0.844) |
| Combined-DT | 0.855 | 0.796 | 0.700 | 0.889 |  | 0.743 | 0.737 | 0.696 | 0.776 |
|  | (0.822-0.887) | (0.743-0.843) | (0.612-0.776) | (0.820-0.934) |  | (0.659-0.820) | (0.646-0.815) | (0.557-0.808) | (0.644-0.871) |
| Combined-L-SVM | 0.821 | 0.759 | 0.692 | 0.822 |  | 0.820 | 0.754 | 0.750 | 0.759 |
|  | (0.774-0.862) | (0.702-0.809) | (0.604-0.769) | (0.745-0.881) |  | (0.758-0.888) | (0.665-0.830) | (0.614-0.852) | (0.625-0.857) |
| Combined-G-SVM | 0.855 | 0.785 | 0.700 | 0.867 |  | 0.790 | 0.772 | 0.750 | 0.793 |
|  | (0.814-0.892) | (0.731-0.833) | (0.612-0.776) | (0.795-0.917) |  | (0.718-0.859) | (0.684-0.845) | (0.614-0.852) | (0.663-0.884) |
| Combined-P-SVM | 0.905 | 0.838 | 0.777 | 0.896 |  | 0.766 | 0.737 | 0.804 | 0.672 |
|  | (0.871-0.934) | (0.788-0.880) | (0.694-0.843) | (0.829-0.940) |  | (0.687-0.835) | (0.646-0.815) | (0.672-0.893) | (0.535-0.786) |

Abbreviations: KNN, k-nearest neighbor; LR, logistic regression; DT, decision tree; L-SVM, linear support vector machine; G-SVM, Gaussian support vector machine; P-SVM, polynomial support vector machine.
